# If the wheel ain’t broke, don’t reinvent it

**DOI:** 10.1186/1476-511X-12-51

**Published:** 2013-04-11

**Authors:** Pauli Ohukainen

**Affiliations:** 1 Institute of Biomedicine, Department of Pharmacology and Toxicology, University of Oulu, Oulu, Finland

**Keywords:** Cholesterol, Mevalonate, Hypothesis

## Abstract

During the past 100 years, several theories explaining human atherosclerosis have been postulated and tested. More than anything else, experimental and observational evidence has supported a very strong causal role for dyslipidemia. This has been established as the current paradigm in both biomedical research as well as public health policies. Recently, a novel hypothesis for the etiology of atherosclerosis was presented. The purpose of this commentary is to critically evaluate its validity.

## Background

In a recent article published in Lipids in Health and Disease [1], a novel hypothesis for the pathogenesis of atherosclerosis is presented. The “mevalonate hypothesis” suggests that stimulation of the mevalonate pathway in endothelial cells and subsequent production of free radicals would be the true cause of both ox-LDL and therefore, also atherosclerosis. Science advances through the constant re-evaluation of existing data but whenever a long-standing scientific consensus is challenged, one is compelled to look very closely at why and how. In this case, the novel hypothesis seems interesting but also unnecessary and critically flawed. The purpose of this commentary is to examine some of the issues of the “mevalonate hypothesis”.

## Discussion

In the article, two main points are raised as being problematic for the prevailing cholesterol theory. One is that ox-LDL is the only thing that really matters in atherogenesis and that its absolute amounts are determined primarily by the concentration of local oxygen radicals. The author suggests that since the amount of these radicals must be low due to their high reactivity, the concentration of LDL itself is irrelevant to the formation of ox-LDL. This premise is flawed in three ways. First, it doesn’t recognize that the widely accepted initiating factor in the pathogenesis of atherosclerosis is actually the retention of apoB-containing particles within the subendothelial proteoglycan [2]. This seems to be the rate-limiting step because if the apoB protein is modified so that it doesn’t bind to proteoglycan, even a marked increase in LDL-C does not promote significant atherogenesis [3]. Secondly, there seems to be several factors besides oxygen radicals that contribute to the oxidation of LDL such as enzymatic reactions [4]. And thirdly, it has been demonstrated that macrophages are able to take up LDL particles even in their unmodified form [5].

The second point raised against the current paradigm is based on lipid-lowering intervention trials. Indeed there have been some negative results but the totality of evidence very strongly suggests that there is substantial benefit in LDL lowering and that this can be seen even when systematically pooling together results from different medications, diets, hormone therapies and even surgical treatments [6]. The author cites some negative statin trials done in highly specific patient populations as examples of the drugs’ universal ineffectiveness. This argument also ignores the totality of evidence that shows that there is indeed benefit from statin therapy and that it is proportional to both the total risk of the patient as well as the absolute reduction in LDL [7]. In addition to these intervention trials, one must not ignore the supporting evidence from low-risk populations [8] and people with lifetime exposure to low LDL due to genetic reasons [9].

All scientific hypotheses can be either validated or falsified by experimental data. If the “mevalonate hypothesis” is true, elevated serum cholesterol is merely a biomarker for increased risk of atherosclerotic disease and not a causal factor. This is contradicted by various experimental animal studies in which exogenous LDL infusions, hypercholesterolemic diet and/or genetic modifications have all been shown to be directly atherogenic. It also seems that the proposed hypothesis is based on the premise that increased cholesterol production in endothelial cells could contribute to serum cholesterol levels. While it is true that accumulation of endogenous cholesterol is toxic to the cell and therefore requires a reverse transport system, this cholesterol efflux from endothelial cells would be reflected only in the HDL fraction of serum cholesterol [10]. There is no evidence that endothelial cells could produce apoB-containing lipoproteins.

## Conclusions

Increased NADPH-oxygenase activity and free radicals probably do have a role in the complex disease that is atherosclerosis. However it seems that there is no real reason to consider a replacement hypothesis for the current paradigm. Also, despite providing food for thought, the suggested “mevalonate hypothesis” is currently unable to overthrow a hundred years’ worth of progressive research.

## Abbreviations

LDL: Low-density lipoprotein; LDL-C: LDL cholesterol; ox-LDL: Oxidized LDL cholesterol; ApoB: Apolipoprotein B; HDL: High-density lipoprotein; NADPH oxidase: Nicotinamide adenine dinucleotide phosphate oxidase.

## Competing interests

The author declared that he has no competing interests.
